# The astrocyte-enriched gene *deathstar* plays a crucial role in the development, locomotion, and lifespan of *D. melanogaster*

**DOI:** 10.1080/19336934.2024.2368336

**Published:** 2024-06-17

**Authors:** Xiaoli Zhang, Dongyu Sun, Kyle Wong, Ammar Salkini, Hadi Najafi, Woo Jae Kim

**Affiliations:** a The HIT Center for Life Sciences, Harbin Institute of Technology, Harbin, China; b Department of Cellular and Molecular Medicine, Faculty of Medicine, University of Ottawa, Ottawa, ON, Canada; c Department of Molecular, Cell and Cancer Biology, University of Massachusetts Chan Medical School, Worcester, MA, USA

**Keywords:** single-cell RNA sequencing, astrocyte, male development, *deathstar*, *CG11000*, *Eaat1*, glia

## Abstract

The *Drosophila melanogaster* brain is a complex organ with various cell types, orchestrating the development, physiology, and behaviors of the fly. While each cell type in *Drosophila* brain is known to express a unique gene set, their complete genetic profile is still unknown. Advances in the RNA sequencing techniques at single-cell resolution facilitate identifying novel cell type markers and/or re-examining the specificity of the available ones. In this study, exploiting a single-cell RNA sequencing data of *Drosophila* optic lobe, we categorized the cells based on their expression pattern for known markers, then the genes with enriched expression in astrocytes were identified. *CG11000* was identified as a gene with a comparable expression profile to the *Eaat1* gene, an astrocyte marker, in every individual cell inside the *Drosophila* optic lobe and midbrain, as well as in the entire *Drosophila* brain throughout its development. Consistent with our bioinformatics data, immunostaining of the brains dissected from transgenic adult flies showed co-expression of *CG11000* with *Eaat1* in a set of single cells corresponding to the astrocytes in the *Drosophila* brain. Physiologically, inhibiting *CG11000* through RNA interference disrupted the normal development of male *D. melanogaster*, while having no impact on females. Expression suppression of *CG11000* in adult flies led to decreased locomotion activity and also shortened lifespan specifically in astrocytes, indicating the gene’s significance in astrocytes. We designated this gene as ‘*deathstar*’ due to its crucial role in maintaining the star-like shape of glial cells, astrocytes, throughout their development into adult stage.

## Introduction

The *Drosophila melanogaster* nervous system is a powerful model to study development [1–4], physiology [4–6], and behaviors [7,8] of other organisms, including human, due to their great homology at cellular and molecular levels [9–12]. The *Drosophila* brain is composed of various cell types which are recognized primarily by their morphology and spatial organization [13–15], hence necessitating the study of this organ deeper at single-cell resolution.

In neuroscience, several classical cell type markers are commonly used to indicate and/or target a particular population of cells, considered to be the cells of a single type [16,17]. At least six main types of neural cells have been identified in *D. melanogaster*, each characterized by the expression of a unique set of genes known as their molecular markers [16]. These cell types include perineurial glia (PNG), sub-perineurial glia (SPG), cortex glia (CG), astrocyte-like glia (ALG or astrocytes), ensheathing glia (EG) and neurons (Neu), each possesses its own genetic driver lines [16].

Since expression pattern of a single or multiple genes could not represent the full genetic profile of a particular cell [18,19], additional analyses are required for cell type identification. Also, besides the previously known molecular markers for each cell type, novel and/or uncharacterized genes may exist in *D. melanogaster* such that a specific cell type can be discerned with. With the advent of single-cell RNA sequencing (scRNA-seq), analysis of the entire transcriptome of an individual cell is feasible, which facilitates determination of the identity of the cells in all organisms including *D. melanogaster*. By providing the full genetic profile of individual cells, scRNA-seq is a convenient strategy to identify novel cell types [20–22] and to re-examine the specificity of the pre-existing molecular markers in the respective cell types [23,24].

In this study, we take the advantages of the availability of the scRNA-seq data of *Drosophila* brain to perform clustering of the sequenced single cells of *Drosophila* brain based on their known molecular markers as well as their entire transcriptome, then seeking for novel genes with differential expression pattern across the clusters. Thereafter, the predicted expression pattern and functional role of the candidate genes are assessed experimentally.

## Materials and methods

### Datasets

A single-cell RNA-sequencing (scRNA-seq) data belonging to the *Drosophila* optic lobe was obtained from the NCBI Gene Expression Omnibus (GEO) database (accession number: GSE103771), which covered the expression profile of 17,272 *Drosophila* genes in a population of 120,000 single cells from optic lobe [25].

Another single-cell RNA-sequencing (scRNA-seq) data was from the *Drosophila* mid-brain and was obtained from the NCBI Sequence Read Archive (SRA) database (accession number: SRP128516), which covered the expression profile of 12,868 *Drosophila* genes in a population of 28,695 single cells from mid-brain [26].

Two bulk RNA-sequencing data of *Drosophila* whole brain were obtained from the NCBI GEO: one contained the expression profile of all *D. melanogaster* genes across four different developmental stages (days 5, 20, 30, and 40 posteclosion; accession number: GSE107049) [27] and another one contained the expression profile of all *D. melanogaster* genes across three different developmental stages of the fly (days 3, 7, and 14 posteclosion; accession number: GSE199164).

### Bioinformatics analyses

In order to have a set of clusters comprising the main neural cell types of *D. melanogaster* (i.e. PNG, SPG, CG, EG, ALG, and neurons), first, the expression matrix belonging to the scRNA-seq data of *Drosophila* optic lobe in the study of Konstantinides et al.. (accession number: GSE103771) [25] was downloaded and used for downstream analyses. Then, this matrix was analyed in accordance with the expression pattern of the previously known *Drosophila* marker genes specific to PNG, SPG, CG, EGN, EGT, ALG, and neuronal cell types and the single cells expressing the markers of a respective cell type and negative for the expression of the markers of other cell types were identified and presented as a heatmap of expression level using the online tool Heatmapper [28] with color codes (red and black colors refer to positive and negative expression for the markers, respectively).

To confirm the above-mentioned cell type classification, hierarchical clustering was performed in accordance with the similarities between the identified single cells based on their transcriptome profile and expressed as a heatmap with color codes for the Pearson correlation coefficient, using the ggplot2 [29] and pheatmap [30] packages in R. Also, principal component analysis (PCA) was performed to distinguish the cells/clusters with distinct transcriptome profile, using R program.

The lists of differentially expressed genes (DEGs) between clusters of interest were identified by the R package limma [31], considering the log_2_fold-change (log_2_FC) ≥ 1 and *P*-value <0.05 as the significance cutoff.

To confirm the differential expression pattern of the candidate genes in a particular cell type/cluster, the unpaired t-test method [32] was applied considering adjusted *P*-values (Benjamini–Hochberg correction) < 0.01 and log_2_FC ≥1 as the significance cutoff. Venn diagram was performed for obtaining the list of common DEGs between the examined clusters, using the web-based tool Venny [33]. Pairwise alignment between the transcripts of *CR34335* and *CR40469* genes was performed in ClustalW [34,35].

### Fly stocks and crosses

All the *D. melanogaster* lines in this study were obtained from the Bloomington Drosophila Stock Center (BDSC) and Vienna Drosophila Resource Center (VDRC). After being subjected to stocks, they were cultured in standard conditions (standard cornmeal-agar yeast fly food at 25°C in a 12 h light:12 h dark condition) following the previous description [36]. CO_2_ served as an anesthetic.

For the crosses of fluorescence labeling experiments, the two binary systems GAL4/UAS-mCD8RFP and LexA/LexAop-mCD8GFP were employed simultaneously in individual male and female flies. The ALG cells of *Drosophila* CNS were labeled in accordance with the expression of a membrane-bound GFP (mCD8GFP) under the control of *Eaat1* gene promoter, fused to the sequence of LexA (*Eaat1-LexA*) (BDSC#: 52719). Transgenic fly lines carrying the *CG11000 (deathstar)-GAL4* were used to drive the expression of a membrane-bound RFP (mCD8RFP) in progenies (both in female and male) under the control of *deathstar* gene promoter (BDSC#: 72713, 23416, 91441). Therefore, the expression pattern of RFP will reflect the pattern of *deathstar* promoter activity across the *Drosophila* single cells. Table 1 lists the fly stocks and the crossing scheme for immunostaining experiments.

**Table 1.** Crossing scheme for imaging experiments of the *Drosophila* brain. The line a contains RFP and GFP reporter genes under the control of UAS and LexAop sequences, respectively. Line B expresses LexA protein in an ALG-specific manner driven by *Eaat1* promoter. The fly line C (of F_1_ generation) was generated by crossing the lines a and B (parents), and then it was crossed to the *CG11000-GAL4* line (i.e. D) to produce F_2_ generation. Among the flies of F_2_ generation, the males and females that lacked balancer chromosomes but contain the complete components of the two binary systems UAS/GAL4 and LexA/LexAop were selected for brain dissection. The females (♀) of all crosses were virgin.

Lines	Fly stocks (genotype & BDSC ID)	Cross scheme
A	*UAS-mCD8RFP, lexAop-mCD8GFP; Sp/CyO; TM2/TM6B* (# 32229)	P: A♀ X B♂ ↓ F1: C♀ X D♂ ↓ F2: E ♂or ♀ ↓
B	*w-; Eaat1-LexA/CyO; TM2/TM6B* (#52719)	
C	*UAS-mCD8RFP, lexAop-mCD8GFP; Eaat1-LexA/CyO; TM2/TM6B* (This study)	
D	*w-; +/+; CG11000-GAL4* (BDSC #72713, 23416, 91441)	
E	*UAS-mCD8RFP, lexAop-mCD8GFP; Eaat1-LexA/+; TM6B/CG11000-GAL4* (This study)	CNS dissection

For functional analysis of the genes, the GAL4/UAS-RNAi system [37] was employed to deplete the function of genes of interest in a cell type-specific manner. Table 2 summarizes the fly stocks (parents) together with their crossing schemes, ensuring progenies to express the *deathstar* RNAi in a cell type-specific manner. In detail, the virgin females transgenic for the *UAS- deathstar RNAi* constructs (BDSC#: 51918, VDRC#: v107504, v102061) were crossed to different male driver lines expressing GAL4 under the control of the promoter of a set of cell type-specific genes (Table 2). The promoter of *shn* gene drives GAL4 expression in PNG, *Mdr65* in SPG, *wrapper* in CG, *CG9657* in EGN, *CG34340* (*Drgx*) in EGT, *CG5264* in ALG, *repo* in all glia, and *nSyb* drives GAL4 expression in neurons [16,23,38]. After crossing the flies, the progenies with complete components of the GAL4/UAS binary system (i.e. *x-GAL4* > *UAS-deathstar RNAi*) were collected based on their phenotypes (Table 2) and functional analyses of *deathstar* gene were performed for them.

**Table 2.** Summary of the crosses and experimental genotypes for behavioral analyses. To generate the transgenic flies with expression of *CG11000-RNAi* in cell type-specific manner, the male flies of different GAL4 driver lines were crossed to the females harboring *UAS-CG11000-RNA*i in their genome (BDSC#: 51918). All the females (♀) for the above-mentioned crosses were virgin. Among the progenies of the above-described crosses, the flies with proper genotypes (harboring both cell type-specific expression of GAL4 and *UAS-CG11000-RNAi*) were selected based on the absence of balancer chromosomes (e.g. *CyO*, *TM3*, and *TM6B*). Three RNAi lines (BDSC# 51918, VDRC# v107504 and VDRC# v102061) have been used to confirm the functional role of *deathstar* gene in astrocytes.

Parents of the crosses (genotype & BDSC or VDRC ID)		Progeny phenotypes (♀ and ♂)	RNAi-expressing cell type
UAS-RNAi transgenic line (♀)	GAL4 driver lines (♂)		
*w*^−^;* +/+; UAS-C11000-RNAi* (# 51918)	w^1118^; +/+; +/+	wild type	-
w^1118^; +/+; +/+	*w*^*1118*^;* +/+; btn-GAL4/TM6B* (#45914)	wild type	-
*w*^−^;* +/+; UAS-C11000-RNAi* (# 51918)	*w*^*1118*^;* +/+; shn-GAL4/TM3* (#40436)	Non-TM3	PNG
	*w*^*1118*^;* +/+; Mdr65-GAL4/TM3* (#50472)	Non-TM3	SPG
	*w*^*1118*^;* +/+; CG-GAL4/TM3* (#45784)	Non-TM3	CG
	*w*^*1118*^;* +/+; CG9657-GAL4/TM3* (#39157)	Non-TM3	EGN
	*w*^*1118*^;* +/+; Drgx-GAL4/TM3* (#39908)	Non-TM3	EGT
	*w*^*1118*^;* +/+; nSyb-GAL4/TM6B* (#68222)	Non-TM6B	Neurons
*w*^−^;* +/+; UAS-C11000-RNAi* (BDSC# 51918, VDRC# v107504, v102061)	*w*^*1118*^;* +/+; btn-GAL4/TM6B* (#45914)	Non-TM6B	ALG

As the control group of the experiment, flies without expression of *deathstar* RNAi but with the same genetic background were used. In fact, the flies of the control group contained the *UAS- deathstar RNAi* construct in their genome but lacked any expression of GAL4 proteins. Thus, no functional RNAi will be produced in this group.

### Immunostaining and imaging

Immunostaining was performed according to the previously-reported protocols [39,40]. In brief, brains were dissected from adults 5 days after eclosion, fixed with 4% formaldehyde for 30 min at room temperature, washed with 1X PBST (1X PBS containing 0.5% Triton-X100) three times (30 min each) and blocked in 5% normal donkey serum for 30 min. The brains were then incubated with primary antibodies in 1X PBST at 4°C overnight, followed by incubation with fluorophore-conjugated secondary antibodies for 1 hour at room temperature. Subsequently, brains were mounted with antifade mounting solution (Invitrogen, catalog number S2828) on slides for imaging.

The primary antibodies were chicken polyclonal anti-GFP (Aves Labs, catalogue number GFP-1010, 1:1000 diluted), rabbit polyclonal anti-RFP (Invitrogen, catalog number 710,530, 1:250 diluted) and mouse monoclonal anti-Bruchpilot (nc82) (DSHB, ID AB-2314866, 1:50 diluted). The applied fluorophore-conjugated secondary antibodies comprised Alexa Fluor 488-conjugated goat anti-chicken (Invitrogen, catalog number A32931, 1:100 diluted), RRX-conjugated donkey anti-rabbit (Jackson Laboratory, catalog number AB-2340613, 1:100 diluted), and Dylight 405-conjugated donkey anti-mouse (Jackson Laboratory, catalogue number AB-2340839, 1:100 diluted). Fluorescence imaging with maximum projections of z-stacks was performed under a confocal laser scanning microscope (Zeiss LSM 7 MP) and the acquired images were processed using the NIH ImageJ [41], and presented by Adobe Illustrator CC 2023 for Mac.

### Colocalization analysis

To perform colocalization analysis of multicolor fluorescence microscopy images, the ImageJ software was used. Initially, the individual channels of the fluorescence image were merged to create a composite view. Then, in the ‘Image’ menu, we selected ‘Type’, and then selected ‘RGB Color’ to ensure the images are displayed in the correct color format. Next, we navigated to ‘Image’, then ‘Adjust’ and selected ‘Color Threshold’, and adjusted the threshold settings to isolate the yellowish pixels indicative of colocalization, and confirmed the selection by clicking the ‘Select’ button. Subsequently, we proceeded to ‘Analyze’, and ‘Measure’ bottons. This action will open a new window displaying the measurement data for the selected area. The ‘Area’ value provided in the measurement window was recorded, as it corresponds to the area of colocalization between the two fluorophores. To calculate the percentage of the colocalized area relative to the total area of the fluorophore of interest (e.g. GFP or RFP), we readjusted the threshold settings to encompass the entire fluorophore area, then clicked on ‘Select’ again. Afterward, we repeated the measurement process by going to ‘Analyze’ and then ‘Measure’ options. This will yield the total area value for the respective fluorophore. Finally, to determine the colocalization percentage, the area values of the colocalized regions were divided by the total area values of total GFP or RFP regions. This calculation provides the colocalization level within the specified region. A representative image of three independent fluorescence imaging experiments with their colocalization regions were then presented. Also, the images were processed with ImageJ software using a threshold function to differentiate fluorescence from background. All specimens were imaged under identical conditions.

### Developmental assay

To assess the possible function of *deathstar* gene in the development of *D. melanogaster* from pupae to adulthood stage, the newly eclosed *D. melanogaster* of the crosses in Table 2 was collected and counted based on their genotypes. After counting, data were presented as the percentage of male and female flies expressing *deathstar* RNAi to the total number of eclosed flies.

### Locomotion assay

We recorded locomotion behaviors of the flies expressing *deathstar* RNAi in astrocytes, and their behaviors were compared to the control group. For this purpose, three different *deathstar* RNAi lines were tested, and the flies without expression of *deathstar* RNAi but with the same genetic background were used as control groups (Table 2). To restrict the expression of RNAi in adult flies, we raised them at 18°C before they eclosed and then transferred them to 29°C following eclosion for locomotion tests. To image fly locomotion, a custom LED array was placed under the stage to serve as backlight. A 2-mm transparent acrylic board sheet was placed on top of the LED array to produce homogeneous illumination. Fly behaviors were recorded using a camera with 1920 × 1080 30 Hz (1080p30). Behavioral arenas were custom built from opaque transparent acrylic board sheets. Chambers were 30 mm in diameter and 2 mm in height. Each fly group was aspirated into a separate chamber and placed on the stage. Flies were observed for 30 min to ensure that no gross motor defects were present before video acquisition was initiated.

### Video acquisition and tracking

Videos were acquired using camera at 1920 × 1080 30 Hz (1080p30). Image segmentation was performed by the custom software Fly Trajectory Dynamics Tracking (FlyTrDT) in python; First, FlyTrDT identifies and quantifies fruit flies into elliptical pixels, and we extracted 2 main features from the videos: 1) The average forward speed and 2) The trajectory map of the fly group in each chamber. For the average forward speed of each group, FlyTrDT records the moving speed of each indoor fruit fly and analyzes the average speed of 10 fruit flies per second. For the trajectory map, FlyTrDT records the movement of each indoor fruit fly, then analyzes and plots the movement trajectories of 10 fruit flies in each chamber, including the movement trajectories of the entire group and the movement trajectories of each fruit fly in the group [42,43].

### Lifespan assay

Lifespan was measured at room temperature according to the standard protocols [44]. In brief, newly ecloded flies (0 to 3 days) were collected (50 per genotype) and then placed in two vials (25 flies per vial), and transferred to fresh vials every two days. Survival was recorded for each genotype (sum of two vials). We scored flies stacked in the food as death events in all the vials analyzed. The survival curves was created with Prism version 10 (GraphPad Software, San Diego, CA, USA) using the method of Kaplan and Meier [45]. For statistical significance of the experiment, one-way ANOVA method was used, and the death rate of each genotype was compared to control group which contained the flies without expression of *deathstar* RNAi but with the same genetic background. *P*-values <0.01 indicated the differences that achieved statistical significance.

### Statistical analysis

Statistical analysis and graphic representations were conducted using Prism 10 for Mac (GraphPad Software, San Diego, CA, USA). Unpaired t-test or one-way ANOVA (using the Bonferroni’s multiple comparison) were applied, which was dependent on the measurements analyzed in the corresponding experiment. The averages with ± SEM in all cases were plotted. *P*-values were determined through two-tailed unpaired Student’s t-test, unless otherwise specified, using GraphPad Prism 10 software. *P < 0.05; **P < 0.01; ***P < 0.001, and ****P < 0.0001.

## Results

### Cell-type identification of single cells in the Drosophila optic lobe

**Figure 1.**
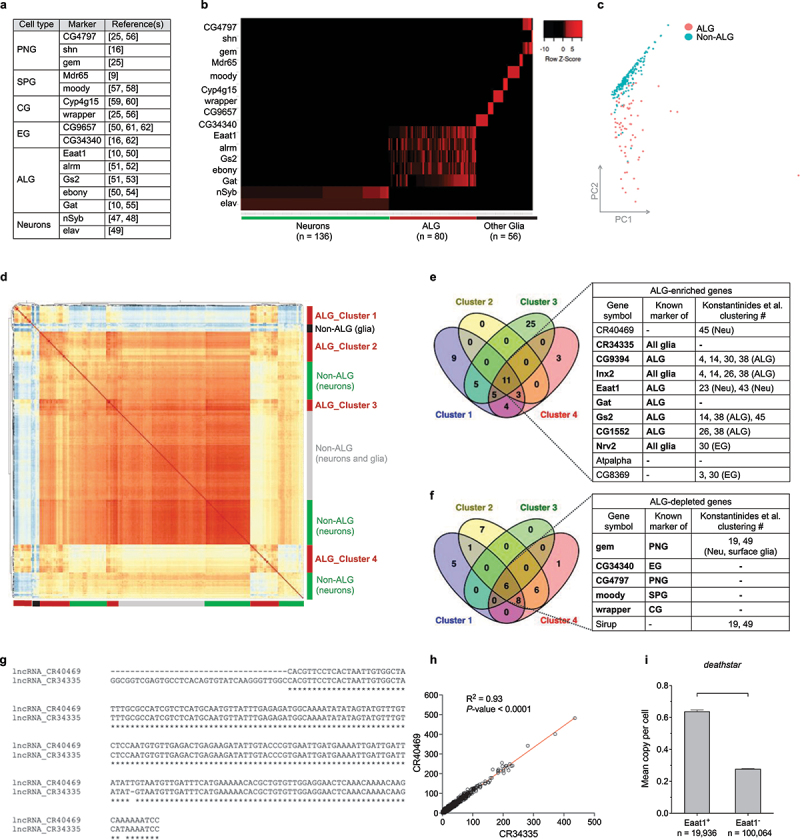
Cell type identification of single cells in *Drosophila* optic lobe. (a) the list of molecular markers used in this study for cell type identification. (b) Classified single cells of *D. melanogaster* optic lobe based on their expression pattern for the markers. Red color denotes ‘expression,’ and black color denotes ‘no expression.’ A row Z-score was used to represent the expression level of each marker. (c) The principal component analysis (PCA) plot distinguishing the ALG single cells (red dots) from single cells of other cell types (labeled as non-ALG, green dots) based on their entire transcriptome profile. (d) Hierarchical clustering categorized ALG cells into four different clusters, all exhibiting distinct global transcriptome profile form other cell types. (e) Venn diagram illustrates the number of the genes upregulated in each ALG cluster. The table lists the common upregulated genes between all ALG clusters. Bold-face fonts represent the genes with cell type-specificity reports in previous studies. If the genes were previously attributed to a particular cell type cluster by Konstantinides et al. [25], the features of the clusters are presented in the table. (f) Venn diagram illustrates the number of downregulated genes in each ALG cluster. The table lists the common downregulated genes in all ALG clusters. Also, the clustering information of Konstantinides et al. study is presented for each gene. (g) Sequence homology between *CR40469* and *CR34335* transcripts using ClustalW. Both sequences are shown in 5‘ → 3’ orientation. (h) Positive expression correlation of *CR40469* and *CR34335* genes in *Drosophila* optic lobe (n = 120,000 single cells). (i) Increased expression of *deathstar* gene in *Eaat1*-expressing (Eaat1^+^) cells in *Drosophila* optic lobe.

Each cell type of *Drosophila* central nervous system is characterized by expression of a unique set of genes, known as their molecular markers [15,16,46]. We used these markers (Figure 1a) to identify the type of single cells in the *Drosophila* optic lobe, sequenced by Konstantinides et al. [25], so that a particular cell type is positive for expression of its markers while negative for expression of the markers of other cell types. Based on these criteria, 156 single cells were identified as neurons, being positive for *nSyb* [47,48] and *elav* expression [49] while negative for all glial markers; 80 single cells were identified as ALG, positive for expression of five astrocytic markers (*Eaat1* [10,50], *alrm* [51,52], *Gs2* [51,53], *ebony* [50,54] and *Gat* [10,55]) and lacking the expression of the markers of other glia and neurons. Also, expression pattern analysis of the markers specific to **PNG**: *CG4797* [25,56], *shn* [16] and *gem* [25], **SPG**: *Mdr65* [9] and *moody* [57,58]), **CG**: *Cyp4g15* [59,60] and *wrapper* [25,56] and **EG**: *CG9657* [50,61,62] and *CG34340* [16,62], has identified 56 single cells, we assigned them all as non-ALG glia or other glia (Figure 1b).

Analysis of the global transcriptome profile of the identified cells through principal component analysis (PCA) illustrated a clear separation of ALG single cells from single cells of other cell types (Figure 1c). Further classification of the identified single cells was performed via hierarchical clustering based on the correlation coefficient of their transcriptome profile, and results illustrated four different clusters for ALG cells (clusters 1–4), three clusters for neuronal cells, and two additional clusters which were mixes of neurons and non-ALG glia (Figure 1d).

To identify the ALG-enriched genes, differential gene expression analysis was performed between each ALG cluster and all clusters of other cell types. Results showed that among all sets of differentially expressed genes (DEGs), the differential expression pattern of 17 genes was common between all ALG clusters, of which eleven genes showed enrichment (Figure 1e), while six genes showed depletion (Figure 1f) in ALG. In fact, the ALG clusters 1 to 4 all showed the enriched expression of some previously known glial markers such as *CR34335* [63], *Inx2* [64–66], and *Nrv2* [23,26,67] and also enriched expression of the astrocyte markers *CG9394* [51], *Eaat1* [25,50,68], *Gat* [50], *Gs2* [25,69], and *CG1552* [51] (Figure 1e; table), while they exhibited the depleted expression of the markers of other cell types (e.g. *gem* and *CG4797* (the PNG markers), *moody* (a SPG marker), *wrapper* (a CG marker), and *CG34340* (a EG marker)) (Figure 1f; table), supporting the identity of the clusters 1 to 4 as astrocytes. The full list of DEGs is presented in Supplemental file S1.

Among the identified ALG-enriched genes (Figure 1e; table), *CR40469* and *Eaat1* were previously classified by Konstantinides et al. in neuronal clusters (clusters # 23, 43, and 45 in their study) [25], while *Eaat1* is a well-characterized marker for astrocytes [6,10,51,68,70,71], and no cell type-specificity was reported yet for *CR40469* gene.

Sequence analysis of *CR40469* transcript (GenBank No: NR_003723.2) demonstrated that it has 96.73% identity with the transcript of the glial marker *CR34335* (GenBank No: NR_133508.1) (Figure 1g), raising the hypothesis that the expression level reported for the *CR40469* in *Drosophila* optic lobe single cells could be influenced by this high-degree sequence homology, hence both transcripts similarly map against *Drosophila* genome leading to highly similar expression counts for both genes. Expression correlation analysis of these genes across all sequenced single cells of *Drosophila* optic lobe (*n* = 120,000 cells) showed a remarkable positive correlation between their expression level (R^2^ = 0.93, P < 0.0001), strengthening this hypothesis (Figure 1h).

*Eaat1* is a known glial marker which is predominantly expressed in astrocytes [72]. To study which gene(s) is/are co-expressed with it, first the sequenced single cells of the *Drosophila* optic lobe [25] were categorized based on their expression pattern for *Eaat1*, then differential gene expression analysis was performed between the cells ‘with’ and ‘without’ *Eaat1* expression (assigned them as Eaat1^+^ and Eaat1^−^ cells, respectively). Results highlighted the differential expression of an uncharacterized gene *CG11000* whose expression was significantly higher in Eaat1^+^ cells (2.43 folds increase, *P*-value <0.0001) (Figure 1i), suggesting potential astrocytic function of this gene (similar to *Eaat1* gene). The full list of the upregulated genes in Eaat1^+^ cells is presented in the supplemental file S2.

### Positive expression correlation of deathstar with Eaat1 in Drosophila brain

**Figure 2.**
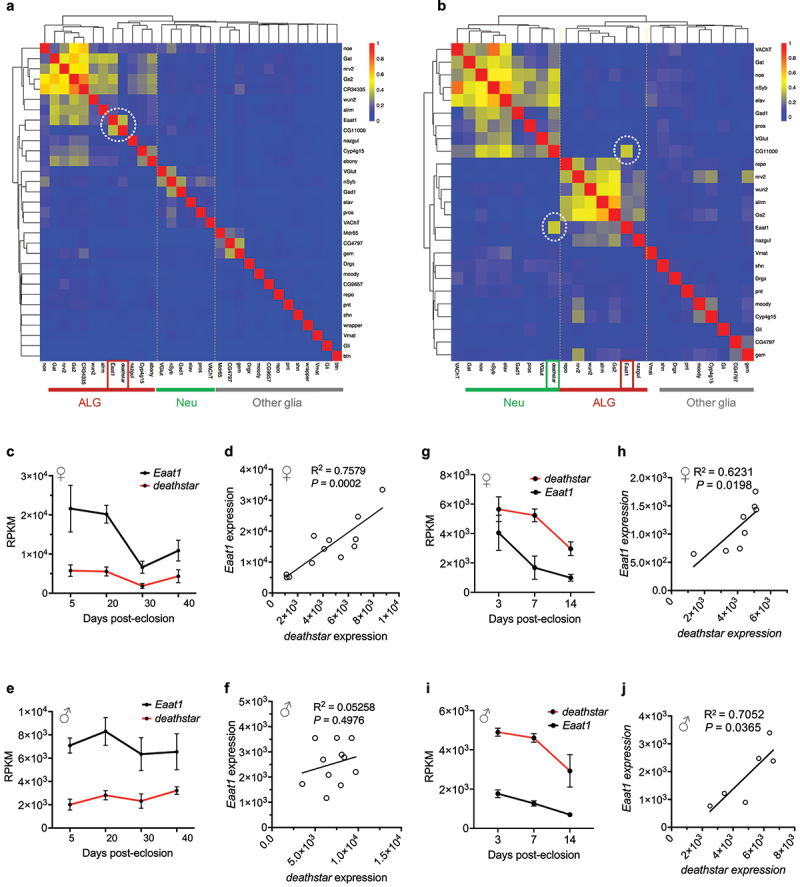
Positive expression correlation of *deathstar* with *Eaat1*. a) Heatmap analysis based on the Pearson correlation coefficient between *deathstar* and known molecular markers of *Drosophila* nervous system across sequenced single cells of *Drosophila* optic lobe (n = 120,000 cells). *deathstar* is co-clustered with the ALG markers including *Eaat1* (dotted circle). b) Heatmap analysis based on the Pearson correlation coefficient between deathstar and known molecular markers of *Drosophila* nervous system across sequenced single cells of *Drosophila* mid-brain (n = 28,695). Two dotted circles in the heatmap point to the positive expression correlation of deathstar with *Eaat1*, among other markers. c-d) Correlation analysis of *deathstar* and *Eaat1* expression level using a bulk RNA-seq data of developing whole brain from female *D. melanogaster* (n = 12 flies). e-f) Correlation analysis of *deathstar* and *Eaat1* expression using a bulk RNA-seq data of developing whole brain of male *D. melanogaster* (n = 11 flies). g-j) Significant expression correlation between *deathstar* and *Eaat1* genes across developmental time-points of female (g-h), and male (i-j) *D. melanogaster*, obtained from the bulk RNA-seq data analysis of developing whole brain of the flies.

The gene *CG11000* was named ‘*deathstar*’ due to its crucial role in determining the fate of star-shaped astrocytes in fruit flies, referencing the iconic space station from Star Wars. The *deathstar* gene is implicated in the regulation of cell differentiation, predicted to be located in the membrane, and expressed in the adult head [73]. Given the increased expression level of *deathstar* gene in Eaat1^+^ cells, its expression pattern was compared to the expression pattern of the previously known cell type markers of *D. melanogaster* across full set of single cells in the *Drosophila* optic lobe. Results showed a positive expression correlation between *deathstar* and *Eaat1* genes, as well as the coclustering pattern of these genes in the resultant heatmap (*n* = 120,000 cells) (Figure 2a). The positive expression correlation of *deathstar* and *Eaat1* genes was also observed in the single cells of *Drosophila* mid-brain (*n* = 28,695 cells), sequenced by Croset et al. [26] (Figure 2b).

Moreover, the expression level of *deathstar* and *Eaat1* genes was examined in a bulk RNA-seq data of developing *Drosophila* whole brain (GSE107049) [27] and results consistently demonstrated a significant positive correlation between the expression level of these genes in females (R^2^ = 0.7579, *P*-value = 0.0002) (Figure 2c,d) and at a nonsignificant level in males (R^2^ = 0.05258, *P*-value = 0.4976) (Figure 2e,f). However, the positive expression correlation of these genes was evident in both females (Figure 2g,h) and males (Figure 2i,j) of an additional bulk RNA-seq data from the developing whole brain of *D. melanogaster* (GSE199164), supporting the positive correlation between expression level of *deathstar* and *Eaat1* genes.

### Coexpression of deathstar and Eaat1 genes in Drosophila brain

**Figure 3.**
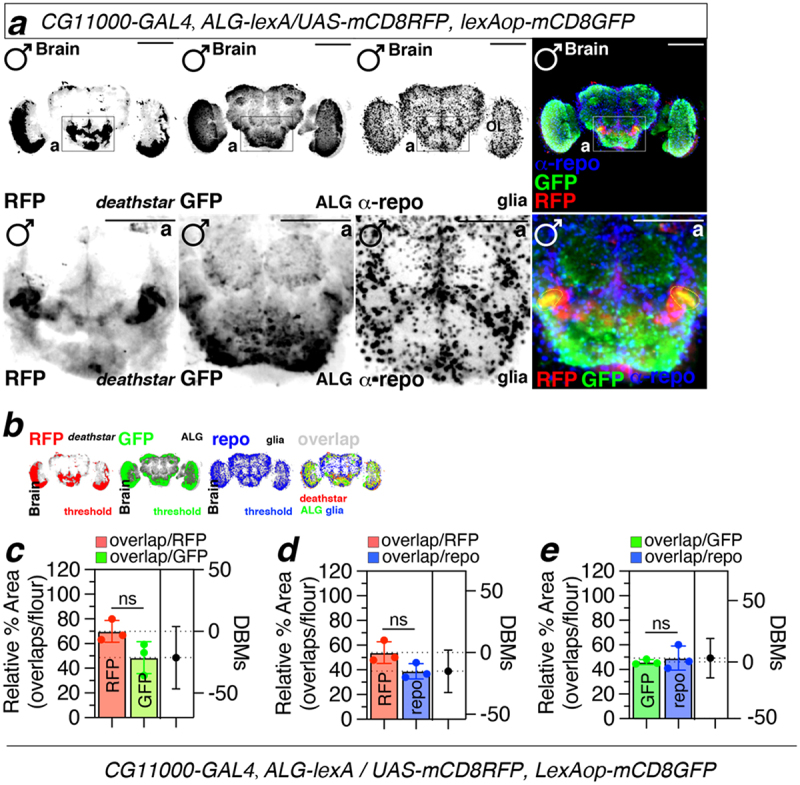
Coexpression of *deathstar* and *Eaat1* in the *Drosophila* brain. a) Overlapping fluorescence signals correspond to the *deathstar* (RFP, red) and *Eaat1* (GFP, green) expression, was shown in the midbrain of *D. melanogaster* (denoted as yellow dotted lines in the lower panel). Anti-repo antibody (α-repo) was used for labeling the glia in the *Drosophila* brain (blue). Scale bars = 50 µm. b) Results of the threshold function in ImageJ to differentiate real fluorescence signals from background for optimal quantification of overlapping fluorescent signals. Red signals denote *deathstar* (BDSC#: 91441), green signals denote *Eaat1* (BDSC#: 52719), and blue signals denote repo expression. c-e) Quantification data of the overlapping signals between *deathstar* and *Eaat1* (c), between *deathstar* and Repo (d), and between Eaat1 and *Repo* genes (e).

To test whether *deathstar* gene is co-expressed with *Eaat1* in single cells of *Drosophila* brain, the driver lines expressing GAL4 and LexA proteins under the control of *deathstar* and *Eaat1* promoters have been used. For this purpose, the male flies expressing GAL4 under the control of *deathstar* promoter and expressing LexA under the control of *Eaat1* promoter were crossed to the virgin females transgenic for both *UAS-mCD8RFP* and *lexAop-mCD8GFP* constructs (Table 1), so that the expression pattern of RFP and GFP in progenies will depict the expression pattern of *deathstar* and *Eaat1* genes, respectively. Results illustrated that the fluorescent signals corresponding to the *deathstar* expression (RFP) were overlapped with the *Eaat1* signals (GFP) in a defined set of single cells in *Drosophila* midbrain (Figure 3a). For this experiment, *Eaat1* and *repo* genes were used for astrocytes (green signals) and all glia (blue signals), respectively (Figure 3b). Quantification of data from three independent experiments showed that 64% of *deathstar* fluorescence signal overlapped with *Eaat1* signals (Figure 3c), suggesting colocalized expression of these genes in *Drosophila* brain. These ALG cells (coexpressing *deathstar* and *Eaat1* genes) comprised an average of 45.7% of all astrocytes in the *Drosophila* brain (Figure 3c). Also, 46% of the *deathstar* signals were overlapped with repo-expressing cells (Figure 3d). This population of glia (co-expressing *deathstar* and *repo* genes) composed an average of 33.9% of all glia in the *Drosophila* brain (Figure 3d). Using another *deathstar*-GAL4 line in an additional fluorescence imaging experiment also showed coexpression of *deathstar* and *Eaat1* genes in a set of ALG cells in the *Drosophila* optic lobe (Supplemental file S4). These data altogether pinpoint a set of ALG single cells in the *Drosophila* brain, coexpressing the *deathstar* and *Eaat1* genes.

### Sex-biased developmental effects of deathstar gene in D. melanogaster

For functional analysis of *deathstar* gene, its expression was suppressed using a specific RNAi expressed in female and male flies in a cell type-specific manner. To this end, virgin females transgenic for *deathstar-RNAi* construct were crossed to a set of male GAL4 driver lines, enabling their progenies to express the *deathstar-RNAi* in different neural cell types of *D. melanogaster*. We took advantage to use recently developed subtype glial-*GAL4* drivers which are feasibly expressed both in larval and adult subtype glial populations to determine which subtypes of glia is responsible for this phenotype [16,74]. The flies with the same genetic background but without *deathstar-RNAi* expression were used as the control group of this experiment (Table 2).

**Figure 4.**
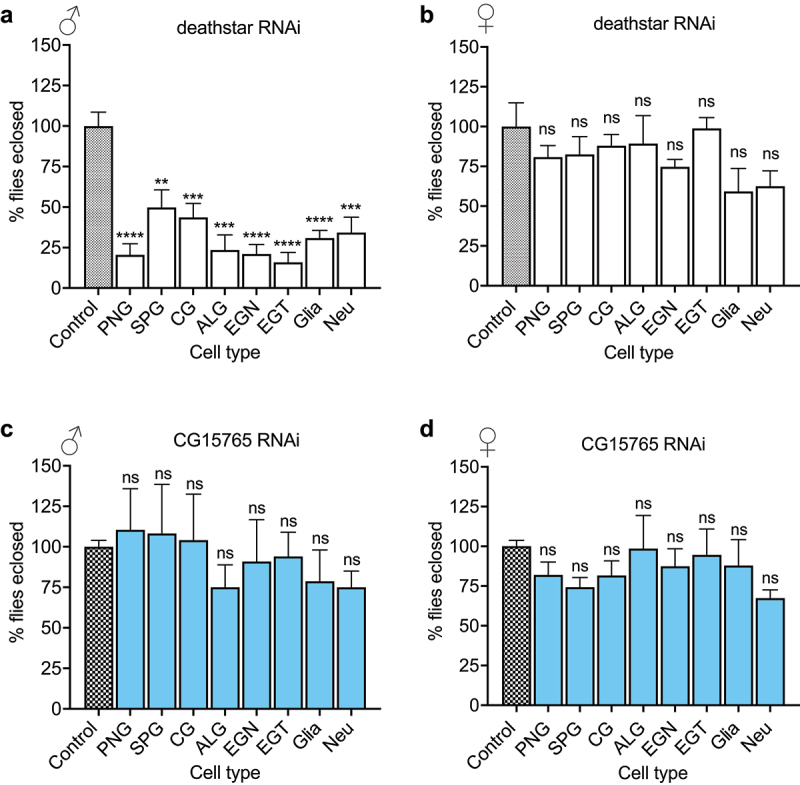
The effects of *deathstar* knock-down on normal development of D. melanogaster. a) In comparison with the control group, the majority of male progenies with *deathstar* knock-down did not develop into adults, indicated by significant reduction in eclosion rate (%). b) *Deathstar* knock-down in females did not show any developmental defects. Differences between the eclosion rates (%) of control group and the progenies with cell type-specific *deathstar* knock-down were not statistically significant (ns = nonsignificant). c-d) Eclosion rate of the progenies expressing an off-target RNAi, targeting the *Drosophila CG15765* gene but not *deathstar*. The eclosion rate (%) was similar for all groups in both males and females.

Through counting the eclosed flies of the crosses, unexpectedly, we observed a sex-biased eclosion rate for the progenies expressing *deathstar-RNAi*, wherein most males expressing *deathstar-RNAi* did not develop into adult flies (Figure 4a), while the females with the same genotypes normally developed into adults (Figure 4b), suggesting a male-specific developmental function for *deathstar*.

A parallel experiment with the same procedure was carried out using an off-target RNAi, which targets the *Drosophila* gene *CG15765* (but not *deathstar*), and the results showed a standard ratio of males over females (~1) for all progenies which expressed *CG15765-RNAi* (Figure 4c,d), supporting the specificity of *deathstar* RNAi to impede development of male progenies.

### Deathstar affects locomotion and lifespan in D. melanogaster

**Figure 5.**
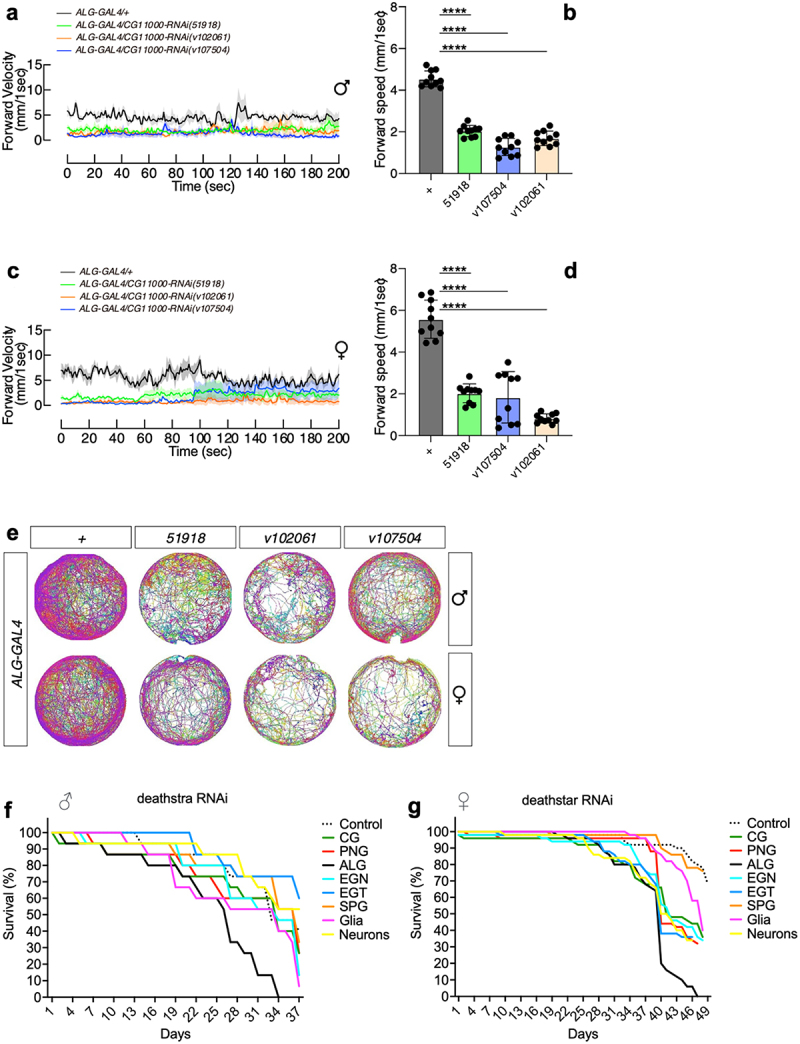
The effects of *deathstar* knock-down on the locomotion and lifespan of adult flies. a) Expression suppression of deathstar in astrocytes of male flies resulted in the decreased level of forward velocity per second, in a 200-second time course, in comparison with the control groups which do not express any RNAi (denoted as grey/black lines). b) Average velocity rate of the flies with suppressed *deathstar* expression in comparison with the control group (*P*-value <0.0001). c) Expression suppression of *deathstar* in astrocytes of female flies resulted in the decreased level of forward velocity per second, in a 200-second time course, in comparison with the control groups which lacked RNAi expression. d) Average velocity rate of the flies with suppressed *deathstar* expression in comparison with the control group (*P*-value <0.0001). e) Trajectory map analysis of the flies in the RNAi groups versus control groups, illustrating impeded locomotion activities in both male and female flies with suppressed expression level of *deathstar* using three different RNAi lines. f–g) ALG-specific reduction of lifespan in both male and female *D. melanogaster*, under expression suppression of *deathstar* gene.

As revealed by this study, the expression level of *deathstar* and *Eaat1* are positively correlated. On the other hand, *Eaat1* is a well-known astrocytic marker whose expression in astrocytes was shown to be critical to locomotion activities in *D. melanogaster* [68]. To investigate if *deathstar* could also affect locomotion in *D. melanogaster*, locomotion assay was performed on adult flies which expressed *deathstar-RNAi* specifically in astrocytes. Results showed that the flies with the expression of *deathstar-RNAi* in their astrocytes had defects in their locomotion activity in comparison with the control group, shown by reduced forward velocity values per second in a 200-second time course (Figures 5a and 5c). Also, the average forward velocity rate of these flies revealed remarkable decreases in the locomotion activity of the flies expressing *deathstar* RNAi in their astrocytes (Figures 5b and 5d), suggesting the crucial role of *deathstar* in astrocytes.

Moreover, trajectory map analysis illustrated the reduced locomotion activity of the flies under suppression of *deathstar* expression in astrocytes, which was confirmed by using different independent RNAi lines (Figure 5e). The astrocyte-specific effects of *deathstar-RNAi* on locomotion behaviors of *D. melanogaster* were identified in both male (Figure 5a,b) and female (Figure 5c,d) flies, suggesting that the function of *deathstar* gene on *Drosophila* locomotion is not sexually dimorphic.

To test the possible function of *deathstar* gene in *D. melanogaster* lifespan, we supressed its expression in different cell types through expressing the *deathstar* RNAi under regulation of cell type-specific GAL4 expression, then their lifespan for 40 ~ 50 days was measured. The most remarkable decreases in the lifespan were identified for the flies expressing the *deathstar-RNAi* specifically in their ALG cells (Figure 5f,g), supporting the astrocytic function of *deathstar* in shortening the lifespan of *D. melanogaster*. The ALG-specific effects of *deathstar-RNAi* on lifespan was also identified in both male (Figure 5f) and female (Figure 5g) flies, suggesting that the function of *deathstar* gene on *Drosophila* lifespan is not sexually dimorphic.

## Discussion

Like other metazoans, the *D. melanogaster* brain is formed by a large number of neurons and glia, each with distinct transcriptome profile conferring them unique structural and/or functional characteristics [63,75]. Cells do not act individually in brain but instead they make several clusters of single cells (known as cell types [10]) with specialized function and/or organization within neural circuits [76–78]. PNG, SPG, CG, EG and neurons are the main well-characterized cell types in the *Drosophila* nervous system [9,15,16,79,80].

Moreover, establishing and maintaining the identity of these cell types throughout the lifespan of the fly require expression of unique set of genes, known as molecular markers [9,15,16,23]. These markers are mostly the genes whose expression implements particular molecular pathways to specialize the development and/or function of the respective cell types [81,82]. In this study, we used the previously known cell type-specific markers (Figure 1a) to classify the sequenced single cells of *Drosophila* optic lobe into their corresponding cell types. At least, two different molecular markers per each cell type were used for this purpose. These markers were among the best well-characterized ones in *D. melanogaster*, whose GAL4/UAS driver lines were also used frequently in previous studies for characterization, mapping and functional analysis of the respective cell types [9,15,16,25,79,80].

On the other hand, availability of the transcriptome profile of *Drosophila* brain at single-cell level enabled us to re-examine the identity of our classified cells, based on their similarities in their entire transcriptome. As the result, the single cells belonging to the ALG cell type (astrocytes) exhibited a far distinctive transcriptome profile versus PNG, SPG, CG, EG and neurons when they were classified based on the expression pattern of their markers (Figure 1b,c). Differential gene expression analysis showed significant enrichment of 11 genes while depletion of 6 genes in the identified ALG clusters (Figure 1e,f). In addition, while most of these identified ALG-enriched genes were among the previously reported ALG-enriched/specific markers, the *Eaat1* and *CR40469* genes were determined as neuronal markers by Konstantinides et al. [25]. These findings led us to focus on these genes which we found them as the top ALG-enriched genes (Supplemental file S1), and also myriad number of previous studies characterized the *Eaat1* as an astrocytic marker [10], and no cell type specificity was reported for *CR40469*, yet.

*CR40469* (also known as *lncRNA:CR40469*) spans a region of 213 kb on the X chromosome of *D. melanogaster* (NCBI GenBank, 2024 update). This gene is known to encode a long noncoding RNA (lncRNA) with 214 nucleotides with unknown function [83–85]. Moreover, due to the high sequence identity rate between *CR40469* gene and the glial marker *CG34335*, functional study of *CR40469* via RNAi-based strategies will be challenging. In a study by Rois et al. [86], a genomic knock-out of this gene in *D. melanogaster* was constructed and through differential gene expression analysis the greatest expression level alterations were found for the genes located on the *Drosophila* chromosome X, nearby the *CR40469* locus (e.g. *CG42259*, *png*, and *CG4313* genes). According to this data, a trans-acting function was attributed to the *CR40469* lncRNA, by which it regulates the expression of its neighboring genes on chromosome X [86]. Here we found that the expression pattern of *CR40469* across the single cells of *Drosophila* optic lobe resembled that of *CR34335* gene which is consistent with previous studies which reported the *CR34335* gene as a glial marker [63]. Also, consistent with our results for ALG-enrichment of *CG40469* lncRNA, it was found by Croset et al. [26] as a glial and astrocyte-enriched gene, suggesting its pivotal role in these cell types. However, further functional studies are required to elucidate the mechanistic role of *CR40469* in its corresponding cell types.

Unlike *CR40469* gene, *Eaat1* is a well-characterized gene crucial for the development, physiology, and behaviors of *D. melanogaster*. When we categorized the sequenced single cells of *Drosophila* optic lobe based on their expression for *Eaat1*, we found the differential expression of an uncharacterized gene, named *CG11000* (we renamed it *deathstar*), which was significantly higher in Eaat1^+^ cells versus the single cells without *Eaat1* expression (Figure 1i). This preliminary data compelled us to investigate the expression correlation of *Eaat1* and *deathstar* genes in *Drosophila* brain. Interestingly, the positive correlation was existed between these genes across the total set of single cells in *Drosophila* optic lobe and mid-brain, as well as across all developmental stages of *Drosophila* whole brain (Figure 2). Beside positive correlation, *deathstar* was clustered in the ALG cells of *Drosophila* optic lobe (Figure 2a), while clustered in the neuronal cluster in the mid-brain (Figure 2b) suggesting some non-ALG expression for this gene. In support of the positive expression correlation of *deathstar* and *Eaat1* genes, their expression level in the single-cell collection of *Drosophila* adult brain was analyzed in SCope database [87], and a significant positive correlation was observed (R^2^ = 0.661, P < 0.0001; supplemental file S3).

Our data also provide a comparative assessment between two approaches of cell type identification: 1-cell type identification based on the predefined set of molecular markers, and 2- cell type identification based on the similarities in whole transcriptome profile.

Since a set of multiple marker genes could not represent the complete genetic profile of a respective cell type, and on the other hand, different cell types may have similar transcriptome profile but differ only in the expression pattern of a limited number of genes, each of the above-mentioned approaches has its own pros and cons that should be considered for cell type identification purposes. For instance, *Eaat1* expression pattern *per se* could not distinguish ALG cells from other cell types since *Eaat1* was shown to be expressed also in a subset of neurons [25]. Similarly, some clusters in the Konstantinides et al. study were identified as neurons based on their entire transcriptome profile (clusters # 4 and 14), while they were positive for the glial marker *repo* [25], which necessitate testing additional markers on these clusters to reveal their actual identity. Also, studies have shown that while Repo protein is a good marker for glial cells [88,89], its transcripts are lowly expressed and often not detected in glial clusters [90], which challenges the use of *repo* gene as a glial marker in RNA-seq data analyses.

In *Drosophila* research, the binary systems such as GAL4/UAS and LexA/LexAop are powerful genetic tools to map the expression pattern of a gene-of-interest against different cell types [14]. Here, we used these systems to trace the distribution of mCD8RFP and mCD8GFP signals across the *Drosophila* optic lobe driven by *deathstar* and *Eaat1* promoters, respectively. The overlapped fluorescent signals of these genes illustrated their co-expression in a set of single cells in *Drosophila* midbrain and optic lobe (Figure 3 and supplemental file S4). However, some single cells in the *Drosophila* brain did not show such co-expression pattern which is consistent with the observed expression pattern of the *deathstar* and *Eaat1* genes in a cluster of single cells in adult *Drosophila* brain obtained from SCope, wherein some single cells express *deathstar* transcripts while negative for *Eaat1* expression (Supplemental file S3).

Functional analysis of *deathstar* revealed a male-specific developmental function of this gene which was not cell type-specific (Figure 4a). Such sex-biased developmental effect of the gene gene could suggest that *deathstar* may exert its functional role in the development of *D. melanogaster* through cellular and/or molecular pathways, which act differently in males and females. Further functional studies are required to elucidate the molecular mechanisms underlying the sex-biased effects of *deathstar* knock-down.

However, *deathstar* knock-down in adult flies resulted in a reduced locomotion activity in both sexes (males and females), suggesting that the effect of *deathstar* gene on locomotion behaviours of the fly is not sexually dimorphic (Figure 5a–e).

Besides changing the locomotion activities, *deathstar* knock-down resulted in shortened lifespan of both males and females in an ALG-specific manner (Figure 5f,g). This result highlights the crucial role of *deathstar* gene in normal lifespan of *D. melanogaster,* which is not sexually dimorphic.

Taken together, this study for the first time delineated the expression pattern and crucial role of the *deathstar* gene in normal development, locomotion and lifespan of *D. melanogaster,* which is an initial step toward further functional characterization of this gene.

## Supplementary Material

Supplemental file S3_May 21 2024.docx

Supplemental file S4_May 21 2024.docx

Supplemental file S2_May 21 2024.xls

Response to the comments_June 8 2024.docx

Supplemental file S1_May 21 2024.xls

## Data Availability

The authors confirm that the data supporting the findings of this study are available within the article and its supplementary materials.
