# Supplementary material for: The astrocyte-enriched gene deathstar plays a crucial role in the development, locomotion, and lifespan of D. melanogaster
Source: Fly (Austin). 2024 Jun 17;18(1):2368336. doi: 10.1080/19336934.2024.2368336 (PMC11185185; doi:10.1080/19336934.2024.2368336)
Supplement: Supplemental file S3_May 21 2024.docx [file KFLY_A_2368336_SM5409.docx]

**Supplemental file S3. Co-expression of *deathstar* and *Eaat1* genes in a set of single cells in adult *Drosophila* brain adopted from SCope.**

**A)** Expression pattern of *deathstar* transcripts. **B)** Expression pattern of *Eaat1* transcripts. **C)** Merged pattern of *deathstar* and *Eaat1* expression across the single cells co-expressing them. **D)** Correlation co-efficient analysis of the expression level of *deathstar* and *Eaat1* genes.
