# Supplementary material for: The astrocyte-enriched gene deathstar plays a crucial role in the development, locomotion, and lifespan of D. melanogaster
Source: Fly (Austin). 2024 Jun 17;18(1):2368336. doi: 10.1080/19336934.2024.2368336 (PMC11185185; doi:10.1080/19336934.2024.2368336)
Supplement: Supplemental file S4_May 21 2024.docx [file KFLY_A_2368336_SM5408.docx]

**
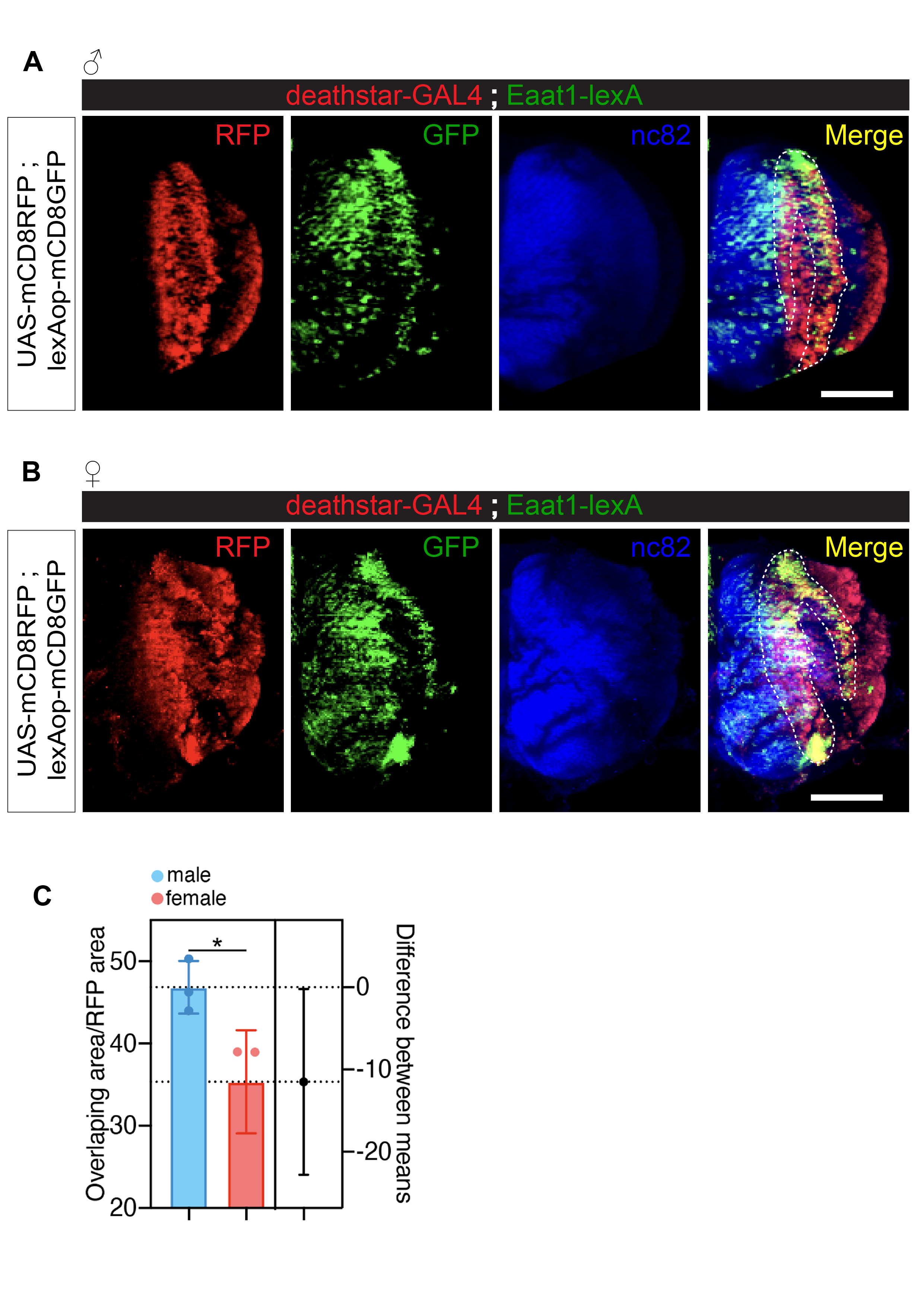
**

**Supplemental file S4.** Co-expression analysis of *deathstar* and *Eaat1* genes in the *D. melanogaster* optic lobe.

**A)** Overlapping fluorescence signals correspond to the *deathstar* (RFP; red) and ALG (GFP; green) expression, tested in the optic lobe of a male *D. melanogaster*.

**B)** Overlapping fluorescence signals correspond to the *deathstar* (RFP; red) and ALG (GFP; green) expression, tested in the optic lobe of a female *D. melanogaster*.

**C)** Percent of overlapping area quantified from the florescent signals in the optic lobes of the tested male and female flies. *P* = 0.0475.
