# Supplementary material for: The astrocyte-enriched gene deathstar plays a crucial role in the development, locomotion, and lifespan of D. melanogaster
Source: Fly (Austin). 2024 Jun 17;18(1):2368336. doi: 10.1080/19336934.2024.2368336 (PMC11185185; doi:10.1080/19336934.2024.2368336)
Supplement: Response to the comments_June 8 2024.docx [file KFLY_A_2368336_SM5406.docx]

**Manuscript number: KFLY-2023-0018R1**

**Corresponding authors:** Hadi Najafi, Woo Jae Kim

**Editor comments.**
Peer-review of your revised manuscript is now complete. The major points of criticism have been satisfactorily fixed. However, Reviewer 1 has identified a few minor corrections that still need to be implemented (see reviewer comments below). Once this is done I shall be pleased to accept your manuscript for publication.
Since it should be a relatively simple task I have set the due date for the final revision as 21 June (15 days from today). If you require more time, please just let me know.
In accordance with our format-free submission policy, an editable version of the article must be supplied at the revision stage. Please submit your revised manuscript files in an editable file format.

Please be sure to append a file detailing, point-by-point, the changes you have made in response to the reviewer comments.

**Answer:** Thank you for your valuable comments and your considerations for publishing our manuscript in the Fly journal. According to the reviewer comments listed below, corrections are made and the updated files (including the updated figure 3 and also the manuscript) are now uploaded in the website of the journal. Also, the point-by-point responses to the reviewer’s comments are provided below.

Sincerely yours,

Hadi Najafi & Woo Jae Kim

**Comments from the Reviewers:**
**Reviewer 1:**
In this revised version of the article "The astrocyte-enriched gene deathstar plays a crucial role in the development, locomotion and lifespan of D. melanogaster ", by Zhang et al, the authors have made significant efforts to address reviewer criticisms including:

-Producing higher resolution and convincing images for staining colocalization and signal quantification, 
-Obtaining consistent and new results with several RNAi and driver lines. Moreover, novel trajectory map analysis with three different RNAi completes adequately the impeded locomotion activities in male and female,
-Developing discussions concerning result variability and statements based on the results

At this point, I only have some minor comments and corrections: 

- Please modify lines UAS-mCD8GFP in the new Fig.3 c, d, e; they should be lexAop-mCD8GFP,

**Answer:** Thank you for this valuable comment. Correction is made and the figure 3 is now updated accordingly.

- Please cite also the number of the GAL4 line (among the three listed in material and methods) used in Fig.3.

**Answer:** Thank you for this valuable comment. The ID number of the GAL4 line used for the experiment in the fig. 3 is now added to the manuscript (line 789).

What does TG4 mean in Fig 3 c-e?

**Answer:** Thank you for your valuable comment. We corrected the ‘TG4’. We are sorry about this typographical error. It should be ‘GAL4’, therefore, it is now corrected in the updated figure 3 file.
